# Development of airflow limitation, dyspnoea, and both in the general population: the Nagahama study

**DOI:** 10.1038/s41598-022-24657-w

**Published:** 2022-11-21

**Authors:** Mariko Kogo, Susumu Sato, Shigeo Muro, Hisako Matsumoto, Natsuko Nomura, Noriyuki Tashima, Tsuyoshi Oguma, Hironobu Sunadome, Tadao Nagasaki, Kimihiko Murase, Takahisa Kawaguchi, Yasuharu Tabara, Fumihiko Matsuda, Kazuo Chin, Toyohiro Hirai

**Affiliations:** 1 Department of Respiratory Medicine, Graduate School of Medicine, Kyoto University, 54 Kawahara-Cho, Shogoin, Sakyo-Ku, Kyoto, 606-8507 Japan; 2 Department of Respiratory Care and Sleep Control Medicine, Graduate School of Medicine, Kyoto University, Kyoto, Japan; 3 Department of Respiratory Medicine, Nara Medical University, Kashihara, Nara Japan; 4 Department of Respiratory Medicine and Allergology, Kindai University, Osakasayama, Osaka Japan; 5 Center for Genomic Medicine, Graduate School of Medicine, Kyoto University, Kyoto, Japan; 6 Graduate School of Public Health, Shizuoka Graduate University of Public Health, Shizuoka, Japan; 7 Department of Sleep Medicine and Respiratory Care, Division of Sleep Medicine, Department of Internal Medicine, Nihon University of Medicine, Tokyo, Japan

**Keywords:** Medical research, Physiology, Respiration

## Abstract

Subjects with subclinical respiratory dysfunction who do not meet the chronic obstructive pulmonary disease (COPD) criteria have attracted attention with regard to early COPD intervention. Our aim was to longitudinally investigate the risks for the development of airflow limitation (AFL) and dyspnoea, the main characteristics of COPD, in a large-scale community-based general population study. The Nagahama study included 9789 inhabitants, and a follow-up evaluation was conducted after 5 years. AFL was diagnosed using a fixed ratio (forced expiratory volume in one second (FEV_1_)/forced vital capacity (FVC) < 0.7). We enrolled normal subjects aged 40–75 years with no AFL, dyspnoea or prior diagnosis of asthma or COPD at baseline. In total, 5865 subjects were analysed, 310 subjects had subclinical respiratory dysfunction (FEV_1_/FVC < the lower limit of normal; n = 57, and FEV_1_ < 80% of the predicted value (preserved ratio impaired spirometry); n = 256). A total of 5086 subjects attended the follow-up assessment, and 449 and 1021 subjects developed AFL and dyspnoea, respectively. Of these, 100 subjects developed AFL with dyspnoea. Baseline subclinical respiratory dysfunction was independently and significantly associated with AFL with dyspnoea development within 5 years. Subjects with subclinical respiratory dysfunction are at risk of developing COPD-like features and require careful monitoring.

## Introduction

Chronic obstructive pulmonary disease (COPD) is an important cause of morbidity and mortality; however, many COPD cases remain undiagnosed globally^1,2^. Undiagnosed early COPD and pre-COPD are associated with poor outcomes^3–6^, therefore, it is necessary to enhance the early identification of COPD^3,4^. In this context, subjects with subclinical respiratory dysfunction who do not meet the COPD criteria of the Global Initiative for Obstructive Lung Disease (GOLD)^7^ have attracted attention. Given the age-related decline in lung function, these subjects may require close monitoring for the development of airflow limitation (AFL). Additionally, the development of respiratory symptoms, which are independent features of COPD, is also of great concern. Particularly, dyspnoea is directly connected to inactivity and can cause a cycle of declining health. Dyspnoea also causes early mortality; therefore, special attention should be paid to this condition^8,9^.

Preserved ratio impaired spirometry (PRISm), characterized by a preserved forced expiratory volume in one second (FEV_1_)/forced vital capacity (FVC) ratio for proportionate impairments in the FEV_1_ and FVC, is a type of subclinical respiratory dysfunction. PRISm can be defined as a FEV_1_ < 80% of the predicted value (%FEV_1_ < 80%) and FEV_1_/FVC ≥ 0.7^6^. Ageing, cigarette smoke exposure, increased systemic inflammation and obesity have been reported to be involved in PRISm^10^. Such patients exhibit aggravated respiratory symptoms, cardiovascular comorbidities and mortality^4,11,12^. Regarding the development of COPD, patients with PRISm presenting with dyspnoea are at risk of a subsequent diagnosis of COPD^13^. However, the clinical impact of asymptomatic PRISm is unclear.

Other types of subclinical respiratory dysfunction, such as FEV_1_/FVC below the lower limit of normal (LLN), defined as the 5th percentile of the predicted value for FEV_1_/FVC, are also important. As FEV_1_/FVC commonly exhibits an age-dependent decline^14^, the definition of AFL based on a fixed ratio of 0.7 could lead to the overestimation of AFL among older subjects and underestimation among younger subjects. Previous studies describing the significance of FEV_1_/FVC < LLN mainly focused on the potential overdiagnosis of AFL based on the use of a fixed ratio, and in a population-based study, higher rates of morbidity and mortality were observed in subjects with FEV_1_/FVC < LLN^15–18^.

We hypothesized that these types of subclinical respiratory dysfunction, including FEV_1_/FVC < LLN and PRISm, together with smoking status and comorbidities could be risk factors for the development of COPD. The specific goal of this study was to identify the risk factors for the development of AFL; respiratory symptoms, particularly dyspnoea; and both.

Clinically, subjects with AFL and dyspnoea could be diagnosed with COPD; therefore, this investigation may contribute to the early detection of COPD in the general population.

## Results

### Characteristics of the included subjects

**Figure 1:**
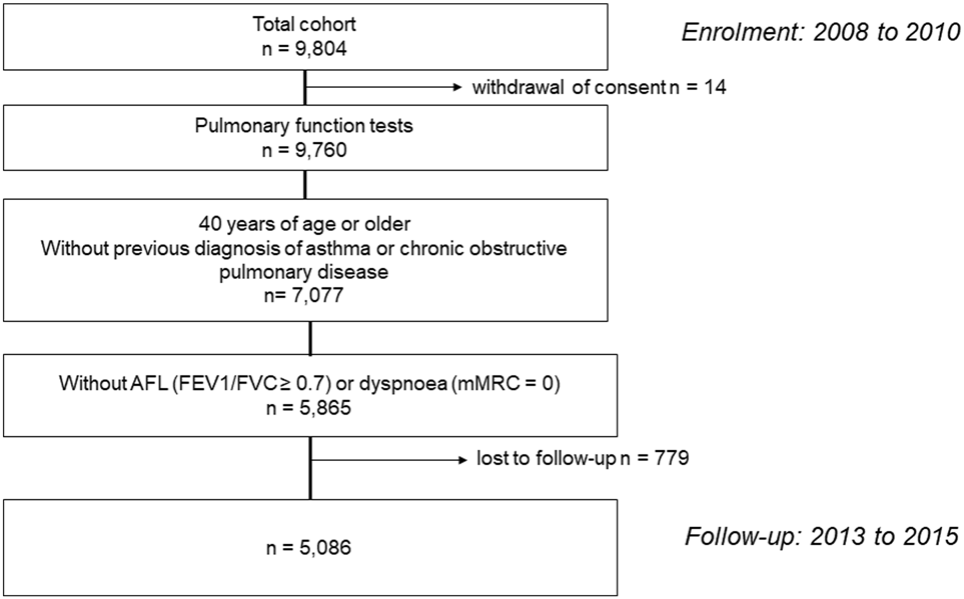
Flowchart of the data extraction process of the Nagahama Study. *AFL* airflow limitation, *FEV*_*1*_ forced expiratory volume in 1 s, *FVC* forced vital capacity, *mMRC* modified Medical Research Council (mMRC) dyspnoea scale.

Of the 9804 participants recruited for the Nagahama study, 5865 individuals aged 40–75 years who did not have AFL (FEV_1_/FVC ≥ 0.7) or dyspnoea (modified Medical Research Council [mMRC] dyspnoea scale = 0) at baseline were included in the current analysis (Fig. 1).

At baseline, 310 subjects had subclinical respiratory dysfunction (based on Japanese predictive equations from the Japanese Respiratory Society (JRS); 57 subjects had FEV_1_/FVC < LLN and 256 subjects had %FEV_1_ < 80%) (Table1). Compared with subjects with normal respiratory function (FEV_1_/FVC ≥ LLN and %FEV_1_ > 80%), subjects with FEV_1_/FVC < LLN were younger (mean 49 years vs. 59 years), were predominantly female (81% vs. 67%), and had a lower body mass index (BMI) (21.4 kg/m^2^ vs. 22.5 kg/m^2^), whereas subjects with %FEV_1_ < 80% (PRISm) were older (62 years vs. 59 years), predominantly male (43% vs. 33%), and more likely to have a smoking history (41% vs. 31%).

**Table 1:** Characteristics of the subjects without AFL or dyspnoea on exertion at enrolment (N = 5865).

	Total	Normal respiratory function*	Subclinical respiratory dysfunction	
			FEV_1_/FVC < LLN	%FEV_1_ < 80%
N	5865	5555	57	256
Age, year	59 (± 9)	59 (± 9)	49 (± 8)^†^	62 (± 9)^‡^
Female, N (%)	3887 (66)	3699 (67)	46 (81)^†^	145 (57)^‡^
Height, cm	159 (± 8)	159 (± 8)	160 (± 8)	160 (± 9)
Weight, kg	57 (± 10)	57 (± 10)	55 (± 10)^†^	59 (± 13)
BMI, kg/m^2^	22.5 (± 3.1)	22.5 (± 3.1)	21.4 (± 2.6)^†^	23.0 (± 3.8)
Smoking status, N (%)	1831 (31)	1713 (31)	13 (23)	105 (41)^‡^
Current	668 (11)	616 (11)	10 (18)	42 (16)^‡^
Former	1163 (20)	1097 (20)	3 (5)^†^	63 (25)^‡^
Pack-years among smokers	12 (± 20)	11 (± 19)	24 (± 23)^†^	17 (± 25)
**Pulmonary function test**				
%FEV_1_, %	104 (± 14)	106 (± 13)	98 (± 14)^†^	73 (± 8)^‡^
%FVC, %	101 (± 14)	102 (± 13)	111 (± 16)^†^	73 (± 9)^‡^
FEV_1_/FVC	0. 82 (± 0.05)	0. 82 (± 0.05)	0.72 (± 0.01)^†^	0.80 (± 0.06)^‡^
**Comorbidities, N (%)**				
Hypertension	1395 (24)	1318 (24)	5 (9)^†^	72 (28)
Diabetes	388 (7)	364 (7)	1 (2)	28 (11)^‡^
Cardiovascular disease	264 (5)	250 (5)	0 (0)	14 (5)

All values are expressed as the mean (± SD) except categorical variables, which are expressed as N (%). *SD* standard division, *FEV*_*1*_ forced expiratory volume in 1 s, *FVC* forced vital capacity, *LLN* lower limits of normal, *BMI* body mass index. *Subjects with FEV_1_/FVC ≥ LLN and %FEV_1_ > 80%. ^†^*P* < 0.05, comparing subjects with FEV_1_/FVC < LLN to those with normal respiratory function. ^‡^*P* < 0.05, comparing subjects with %FEV_1_ < 80% to those with normal respiratory function.

After 5 years, 5086 subjects underwent follow-up assessments (Fig. 1).

The baseline characteristics of the subjects (N = 779) who were lost to follow-up are presented in Supplementary Table S1.

### Development of AFL and dyspnoea

Among the 5086 subjects who attended the 5-year follow-up, AFL was newly identified in 449 subjects (9%); 1021 subjects (20%) had newly developed dyspnoea (mMRC ≥ 1), and 100 subjects developed both AFL and dyspnoea concurrently (AFL with dyspnoea).

Compared with subjects without AFL or dyspnoea at follow-up, subjects who developed AFL were older (mean 63 years vs. 58 years), more likely to be male (55% v 31%), and more likely to smokers (current or former) (49% vs. 28%) at baseline (Table 2), while subjects who developed dyspnoea were older (61 years vs. 58 years), had a higher BMI (23 kg/m^2^ vs. 22.3 kg/m^2^), and were more likely to be current smokers (13% vs. 9%). At follow-up, a higher prevalence of comorbidities, especially hypertension, was observed in both AFL patients and dyspnoea patients than in normal controls (29%, 30% and 21%).

**Table 2:** Characteristics at enrolment of subjects who had AFL, dyspnoea or both at follow-up (N = 5086).

	AFL at follow-up	Dyspnoea at follow-up	AFL with dyspnoea at follow-up	Normal at follow-up
N	449	1,021	100	3716
Age, years	63 (± 8)*	61 (± 9)*	64 (± 8)*	58 (± 9)
Female, N (%)	202 (45)*	702 (69)	50 (50)*	2564 (69)
Height, cm	162 (± 8)*	158 (± 8)	161 (± 8)*	159 (± 8)
Weight, kg	59 (± 10)*	58 (± 10)*	59 (± 11)*	57 (± 10)
BMI, kg/m^2^	22.4 (± 2.8)	23 (± 3.3)*	22.8 (± 3.3)	22.3 (± 2.9)
Smoking status, N (%)	219 (49)*	307 (30)	48 (48)*	1052 (28)
Current	98 (22)*	131 (13) *	27 (27)*	328 (9)
Former	121 (27)*	176 (17)	21 (21)	724 (19)
**Pulmonary function test**				
%FEV_1_, %	96 (± 14)*	103 (± 15)*	94 (± 14)*	106 (± 14)
%FVC, %	99 (± 16)*	99 (± 15)*	98 (± 15)*	102 (± 14)
FEV_1_/FVC	0.76 (± 0.04)*	0.82 (± 0.05)*	0.76 (± 0.04)*	0.83 (± 0.05)
**Comorbidities, N (%)**				
Hypertension	128 (29)*	303 (30)*	30 (30)*	792 (21)
Diabetes	31 (7)	72 (7)	6 (6)	229 (6)
Cardiovascular disease	23 (5)	77 (8)*	11 (11)*	133 (4)

All values are expressed as the mean (± SD) except categorical variables, which are expressed as N (%). *SD* standard division, *AFL* airflow limitation, *BMI* body mass index, *FEV*_*1*_ forced expiratory volume in 1 s, *FVC* forced vital capacity. *P < 0.05, compared with normal subjects without AFL or dyspnoea at follow-up.

Subjects with AFL with dyspnoea were older (64 years vs. 58 years), more likely to be male (50% vs. 31%) and more likely to be current smokers (27% vs. 9%) than normal subjects. They also had higher prevalence rates of hypertension and cardiovascular disease (30% vs. 21% for hypertension and 11% vs. 4% for cardiovascular diseases).

**Figure 2:**
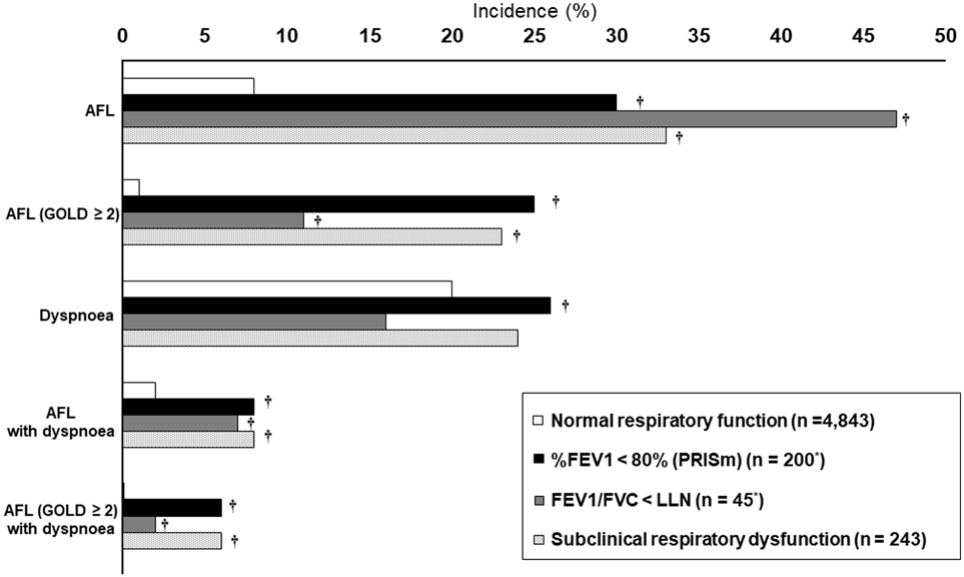
Incidence of the development of AFL, dyspnoea and both among subjects who underwent follow-up assessment. Incidence rates of AFL, dyspnoea, and both (AFL with dyspnoea) at follow-up in groups with normal respiratory function and subclinical respiratory dysfunction at enrolment (N = 5086). A GOLD grade ≥ 2 was defined as the development AFL with %FEV1 < 80%. *AFL* airflow limitation, *FEV*_*1*_ forced expiratory volume in 1 s, *FVC* forced vital capacity. *Two subjects had both FEV_1_/FVC < LLN and %FEV1 < 80% at baseline. ^†^*P* value < 0.05 compared with normal respiratory function.

Figure 2 shows the incidence rates of the development of AFL, dyspnoea, and both at follow-up, according to baseline spirometry characteristics (PRISm, FEV_1_/FVC < LLN, and any “subclinical respiratory dysfunction”). Subjects with any subclinical respiratory dysfunction at baseline had higher incidence rates of AFL (30%, 47% and 33%) and AFL with dyspnoea (8%, 7% and 8%) than those without them. Regarding the development of dyspnoea, those with PRISm at baseline had a higher incidence than normal subjects.

The risk factors for the development of AFL (Table 3A), dyspnoea (Table 3B) and AFL with dyspnoea (Table 4) in the univariate and multivariate analyses are shown. Age, current smoking, cardiovascular disease, PRISm and FEV_1_/FVC < LLN at baseline were significantly associated with the development of AFL with dyspnoea in the multivariate analysis (risk ratio [95% confidence interval]; 1.99 [1.49–2.67] and 2.71 [1.44–5.09], respectively).

**Table 3:** Risks associated with the development of AFL (A) and dyspnoea (B) at 5 years.

(A)	RR^b^	Adjusted RR^b^	(B)	RR^b^	Adjusted RR^b^
Age ≥ 60 years	2.24 (1.84–2.74)	1.58 (1.40–1.79)	Age ≥ 60 years	1.41 (1.26–1.58)	1.22 (1.13–1.31)
Female	0.39 (0.33–0.47)	0.66 (0.57–0.77)	Female	1.07 (0.95–1.21)	1.19 (1.06–1.32)
Smoking status			Smoking history		
Current vs. former	1.54 (1.20–1.97)	2.04 (1.45–2.87)	Current vs. former	1.41 (1.15–1.72)	1.79 (1.37–2.34)
Former vs. never	1.86 (1.51–2.29)	1.06 (0.76–1.48)	Former vs. never	0.82 (0.75–1.01)	0.93 (0.73–1.19)
BMI ≥ 25 kg/m^2^	0.77 (0.61–1.00)	0.76 (0.65–0.88)	BMI ≥ 25 kg/m^2^	1.39 (1.23–1.57)	1.21 (1.11–1.32)
Hypertension	1.31 (1.07–1.59)	1.08 (0.95–1.22)	Hypertension	1.38 (1.23–1.55)	1.14 (1.05–1.24)
Diabetes	1.08 (0.76–1.53)	0.82 (0.67–1.02)	Diabetes	1.13 (0.92–1.39)	0.99 (0.85–1.14)
Cardiovascular disease	1.19 (0.80–1.76)	0.95 (0.75–1.21)	Cardiovascular disease	1.79 (1.48–2.16)	1.43 (1.22–1.69)
%FEV_1_ < 80%	3.74 (2.96–4.73)	2.17 (1.77–2.67)	%FEV_1_ < 80%	1.27 (0.99–1.63)	1.15 (0.96–1.37)
FEV_1_/FVC < LLN	5.51 (3.98–7.63)	4.61 (3.00–7.06)	FEV_1_/FVC < LLN	0.77 (0.39–1.53)	0.96 (0.64–1.43)
SP-370 at follow-up^a^	2.28 (1.61–3.22)	1.58 (1.31–1.90)	SP-370 at follow-up^a^	1.08 (0.92–1.27)	1.03 (0.94–1.14)

*RR* risk ratio, *AFL* airflow limitation, *BMI* body mass index, *FEV1* forced expiratory volume in 1 s, *FVC* forced vital capacity, *LLN* lower limits of normal. ^a^Spirometer used at follow-up. ^b^RR (95% confidence interval).

**Table 4:** Risks associated with the development of AFL with dyspnoea at 5 years.

	RR^b^	Adjusted RR^b^
Age ≥ 60 years	2.98 (1.86–4.77)	1.72 (1.34–2.21)
Female	0.48 (0.32–0.71)	0.84 (0.63–1.11)
**Smoking status**		
Current vs. former	2.54 (1.44–4.49)	3.28 (1.79–6.02)
Former vs. never	1.37 (0.82–2.28)	0.90 (0.46–1.74)
BMI ≥ 25 kg/m^2^	1.10 (0.68–1.79)	0.97 (0.75–1.25)
Hypertension	1.42 (0.93–2.17)	1.07 (0.85–1.34)
Diabetes	0.94 (0.42–2.14)	0.76 (0.50–1.15)
Cardiovascular disease	2.74 (1.48–5.05)	1.54 (1.10–2.15)
%FEV_1_ < 80%	3.73 (2.12–6.57)	1.99 (1.49–2.67)
FEV_1_/FVC < LLN	3.50 (1.15–10.6)	2.71 (1.44–5.09)
SP-370 at follow-up^a^	2.38 (1.11–5.11)	1.47 (1.01–2.15)

*RR* risk ratio, *AFL* airflow limitation, *BMI* body mass index, *FEV1* forced expiratory volume in 1 s, *FVC* forced vital capacity, *LLN* lower limits of normal. ^a^Spirometer used at follow-up. ^b^RR (95% confidence interval).

The associations between subclinical respiratory dysfunction and the development of AFL with dyspnoea were consistent in those with GOLD stage 2 or higher AFL (%FEV_1_ < 80% and FEV_1_/FVC < 0.7) (see Supplementary Table S2). Classifying FEV_1_/FVC based on the LLN defined by the European Respiratory Society Global Lung Function Initiative (GLI) produced results similar to those obtained using the LLN defined by the JRS (see Supplementary Table S3).

Serum brain natriuretic peptide (BNP) was also analysed (see Supplementary note). High BNP was associated with the development of AFL (risk ratio [95% confidence interval]; 1.71 [1.30–2.26]), dyspnoea (1.36 [1.13–1.64]) and AFL with dyspnoea (2.10 [1.19–3.73]) and it still had the positive risks for dyspnoea and AFL with dyspnoea in the multivariate general linear models (see Supplementary Table S4).

## Discussion

We investigated a population-based cohort with follow-up assessments to evaluate both respiratory symptoms and pulmonary function and found that subclinical respiratory dysfunction, represented by FEV_1_/FVC < LLN and %FEV_1_ < 80% (PRISm), was independently associated with the development of AFL, especially AFL with dyspnoea, which is the most important COPD-like feature. Given the need to promote the early detection of COPD, the major finding of the present study is that subjects with subclinical respiratory dysfunction should be observed closely for the development of respiratory symptoms. Moreover, our results revealed the independent impacts of current smoking on the development of all AFL, dyspnoea, and AFL with dyspnoea; therefore, smoking cessation should be encouraged. We also showed that comorbidities and obesity independently contributed to the development of dyspnoea, and a history of cardiovascular disease had an impact on the development of AFL with dyspnoea.

Although the clinical importance of subclinical respiratory dysfunction has been identified, significant associations have been reported with only progression to COPD^10,12,13,19–21^. Park et al. reported that PRISm in elderly patients or those with existing respiratory symptoms was associated with an increased risk of a physician diagnosis of COPD within 3 years^13^. However, their study was limited to smokers, and they established only COPD, not the development of respiratory symptoms (dyspnoea) or AFL, as the outcome. Consequently, the relationship between PRISm and the development of COPD is ill-defined, especially in never smokers and those who are not yet symptomatic. Moreover, to our knowledge, no study has assessed the association between FEV_1_/FVC < LLN but ≥ 0.7 and the development of COPD. The present study builds on previous research on subclinical respiratory dysfunction by clarifying the risks for the development of AFL with dyspnoea at 5 years, which were two- and threefold higher in those with PRISm and FEV_1_/FVC < LLN, respectively, than in those with normal respiratory function, independent of age or smoking status.

The subjects with PRISm who developed AFL or AFL with dyspnoea mostly had GOLD stage 2 or higher disease (Fig. 2), independent of age, smoking status, or a history of cardiovascular disease. We also found that increased serum BNP was associated with both AFL and dyspnoea. These results were in accordance with those of a study on coronary risk factors for COPD^22,23^. Together with the heterogenetic characteristics of PRISm^10^, our results suggest that PRISm in those with a cardiovascular burden requires special attention.

The PRISm patients and control subjects had comparable BMI values, which were much lower than those reported in Western populations^12^. A similar difference in COPD patients between Japanese and Western populations has been reported^24^. Differences in several factors, including ethnicity, genetics, environment, lifestyle, and socioeconomic status, have been considered explanations^24^. These factors could also contribute to the difference in the PRISm rate between the populations. Additionally, the specific causes of low FVC in Japanese PRISm patients could differ from those in Western patients (old tuberculosis for example). The heterogeneity of PRISm should be considered when interpreting our results. Nevertheless, this study is significant in that it suggests that patients with PRISm, regardless of their symptoms or smoking status, could be candidates for early COPD detection in the Japanese population.

Interestingly, the characteristics of subjects with FEV_1_/FVC < LLN in our study were different from those with PRISm; they were younger, were predominantly female, and had a lower BMI than those with normal respiratory function, though the proportions of smokers were similar. Although these characteristics were also different from the characteristics of patients with COPD^25^, they were consistent with those in previous studies in subjects with FEV_1_/FVC between 0.7 and the LLN^19,26^. Impaired lung development is considered a factor affecting low FEV_1_/FVC^19^. The lung function trajectory is characterized by the attainment of maximal pulmonary function, as assessed by FEV_1_, at the age of 20–25 years and a subsequent decline with ageing^27^. Impaired lung development can lead to lower peak lung function and an accelerated loss of function, which are associated with future AFL^28^.

In our study, 9% and 2% of subjects developed AFL and AFL with dyspnoea within 5 years, respectively. These proportions were lower than those in a Western report^12,13^. This might reflect the larger population of never smokers in Japan. Indeed, a Japanese population-based study reported an incidence of AFL similar to that in our study^29^. We acknowledge potential bias from loss to follow-up. However, given the lower FEV_1_ in subjects who were lost to follow-up than in those who were followed, including the lost subjects would increase the incidence of COPD in the group with subclinical respiratory dysfunction. This supports our conclusion that subclinical respiratory dysfunction is a risk factor for the development of COPD.

We observed that different factors were associated with the development of AFL and dyspnoea. Age, male sex, current smoking, low BMI, and subclinical respiratory dysfunction were associated with the development of AFL. However, age, female sex, comorbidities, and obesity were associated with the development of dyspnoea. These differences were previously reported in a cross-sectional study on preclinical COPD^5^. In our study, we found the important contribution of obesity to the development of dyspnoea, despite several studies reporting controversial results and mechanisms of the association between dyspnoea and obesity^3,30–33^. Additionally, in our study, a history of cardiovascular disease was independently associated with not only the development of dyspnoea but also the development of AFL with dyspnoea. Although the mechanism is unclear, previous studies reporting the association between cardiovascular disease and COPD suggested the contribution of pulmonary vascular congestion or proinflammatory molecules, including angiotensin 2, in cardiovascular disease to the development of AFL^22^. On the other hand, there is a possibility that cardiovascular disease itself exacerbates dyspnoea during the process of airway remodelling in COPD. However, AFL, symptoms, and comorbidities are so closely linked in COPD that the GOLD document emphasize the importance of comprehensive management rather than addressing them as separate phenomena. Considering the previous study that found that dyspnoea itself is a risk factor for the development of COPD as well as a cause of morbidity^13^, our results are important in terms of identifying the population at high risk for COPD.

Significant associations between current smoking but not former smoking at enrolment and the development of dyspnoea and AFL with dyspnoea in 5 years were observed. This was previously suggested in a cross-sectional study that showed an association of current smoking but not pack years with severe respiratory symptoms^3^. Our study additionally revealed the importance of smoking cessation in subjects without AFL or dyspnoea in terms of prevention of both COPD and related morbidity.

Numerous clinical trials on the prognostic prediction of COPD have used the GOLD criteria^25,34^, and AFL with dyspnoea in this study comes close to fulfilling those criteria. We believe that identifying subjects at risk of the development of AFL with dyspnoea would be of great benefit in the real world. We found that those with subclinical respiratory dysfunction, including PRISm and FEV_1_/FVC < LNN, were 2- and 3-times more likely than those with normal function to develop AFL with dyspnoea, respectively. We suggest that more attention should be given to these subjects.

A strength of this study is its evaluation of risk factors using a large longitudinal population-based cohort and the 87% follow-up rate. However, some limitations should be mentioned. First, we had access to only spirometry results without bronchodilation. Therefore, we could not fully exclude subjects with reversible AFL. However, several studies have employed pulmonary function test parameters without bronchodilation as a metric^1,35–37^. Additionally, the GOLD guidelines accept this as a pragmatic approach to the identification of cases of COPD^38^. For these reasons, we are confident in our results with regard to the identification of risk factors for the development of COPD. Second, there are no standard reference equations for the Japanese population that are internationally accepted. Therefore, we applied the prediction equations from the JRS to identify FEV_1_/FVC < LLN in the present study. Additionally, we validated our results with GLI-derived reference equations for “other” ethnicities^39^. Third, we evaluated dyspnoea considering only the mMRC criteria. This unidimensional estimation of dyspnoea could cause underestimation and limit the interpretation of our study. However, several studies have described mMRC-defined dyspnoea to be a predictive factor for disability and mortality^9,40^. Therefore, we consider that our study is still of great importance for identifying subjects who may require medical intervention. Additionally, considering that those with an mMRC grade of 1 can be highly symptomatic^40,41^, we used a grade of 1 instead of 2 for the cut-off of the mMRC grade. This minimized underestimation and was suitable for our purpose of promoting early identification of subjects at risk.

We revealed that individuals with subclinical respiratory dysfunction, including FEV_1_/FVC < LLN and %FEV_1_ < 80% (PRISm), are at risk of developing COPD in 5 years. Patients with comorbidities and obesity could develop dyspnoea via mechanisms other than the progression of AFL.

## Methods

### Study design and subjects

This was a population-based observational study based on the Nagahama Cohort for Comprehensive Human Bioscience (the Nagahama Study); subjects from the general population of Nagahama in Shiga Prefecture, Central Japan, were enrolled from November 2008 to November 2010. Residents aged 30–74 years who were able to live independently and lacked serious health or physical impairment were recruited. The participants in this cohort were invited to participate in a follow-up assessment 5 years after enrolment, from 2013 to 2015.

All clinical measurements, pulmonary function tests and blood sampling were performed at enrolment and follow-up. Medical histories were investigated using a structured questionnaire. Dyspnoea was identified using the mMRC criteria^7^, and participants with an mMRC grade of 0 were considered to be free from dyspnoea.

Smoking status was classified as current, former, or never smoker. Cardiometabolic comorbidities, including hypertension, diabetes, and cardiovascular disease (a history of heart disease or stroke)^42,43^, were defined by the responses to the self-reported questionnaires and/or the results of blood tests (fasting blood glucose level ≥ 126 mg/dl, random serum glucose level ≥ 200 mg or HbA1c ≥ 6.5% for the diagnosis of diabetes)^18^.

Among the 9804 residents recruited from 2008 to 2010, 5868 subjects aged 40–75 years who did not have a history of adult asthma or COPD and who did not have AFL (FEV_1_/FVC ≥ 0.7) or dyspnoea (mMRC grade of 0) at the time of enrolment were included (Fig. 1).

This study adhered to the principles of the Declaration of Helsinki. All study protocols were approved by the ethics committee of Kyoto University Graduate School of Medicine and the Nagahama Municipal Review Board (Registry ID G0278). Written informed consent was obtained from all participants.

### Pulmonary function tests

Pulmonary function was measured during an FVC manoeuvre with an electronic spirometer with automated quality checks (baseline; SP-350 COPD, Fukuda Denshi, Tokyo, Japan). In 15% of the subjects, the same type of spirometer (SP-350) was used to measure FVC at baseline and at follow-up, while a different type of spirometer (SP-370) was used in the remaining subjects.

An FVC manoeuvre was performed more than twice at baseline and at follow-up by trained and certified medical technologists to minimize the influence of incomplete effort. The most relevant data was selected by pulmonologists for analysis. AFL was defined based on a fixed ratio (FEV_1_/FVC < 0.7). Predicted normal values for FEV_1_, FVC and the LLN for FEV_1_/FVC were calculated using the JRS guidelines^44^. The LLN defined by the GLI was also used to confirm our results^39^.

### Statistical analysis

The Wilcoxon rank-sum test and Pearson chi-square test were used to compare the characteristics of subjects with and without subclinical respiratory dysfunction at baseline and who had or had not developed AFL or dyspnoea at follow-up. To assess the adjusted risk ratio of the development of AFL, dyspnoea (mMRC ≥ 1) and AFL with dyspnoea in 5 years, we used multivariate general linear models with a Poisson distribution and log link function, with adjustment for age, sex, BMI, smoking history, and major comorbidities. Regarding age and BMI, clinically relevant cut-off values (age ≥ 60 years and BMI ≥ 25) were applied^30^.

A two-tailed *P*-value < 0.05 was considered statistically significant. All statistical analyses were performed using JMP Pro 14 (SAS Institute, Inc., Cary, NC). Data are presented as means (± standard deviations [SDs]) for continuous variables and percentages for categorical variables.

### Ethics approval and consent to participate

This study adhered to the principles of the Declaration of Helsinki. All study protocols were approved by the ethics committee of Kyoto University Graduate School of Medicine and the Nagahama Municipal Review Board (Registry ID G0278). We obtained written informed consent from all the participants.

## Supplementary Information

Supplementary Information.

## Data Availability

The datasets used and/or analysed during the current study are available from the corresponding author upon reasonable request.
