# Supplementary material for: Development of airflow limitation, dyspnoea, and both in the general population: the Nagahama study
Source: Sci Rep. 2022 Nov 21;12:20060. doi: 10.1038/s41598-022-24657-w (PMC9681883; doi:10.1038/s41598-022-24657-w)
Supplement: Supplementary file 1 — Supplementary Information. [file 41598_2022_24657_MOESM1_ESM.docx]

**Supplementary Information**

**Title: The development of airflow limitation, dyspnoea, and both in the general population: The Nagahama study.**

**Authors:**

Mariko Kogo MD; Susumu Sato MD, PhD; Shigeo Muro MD, PhD; Hisako Matsumoto MD, PhD; Natsuko Nomura MD; Noriyuki Tashima MD; Tsuyoshi Oguma MD, PhD; Hironobu Sunadome MD, PhD, Tadao Nagasaki MD, PhD, Kimihiko Murase MD, PhD; Takahisa Kawaguchi PhD; Yasuharu Tabara PhD; Fumihiko Matsuda PhD; Kazuo Chin MD, PhD; Toyohiro Hirai MD, PhD

**Supplementary Note**

Among 5,086 subjects who underwent follow-up assessments, information on serum BNP at baseline was available for 5,085 subjects. The mean (±SD) BNP concentration was 17.9 (±17.0). A high BNP concentration (cut-off value of 40 pg/mL^1^) was associated with the development of all AFL (risk ratio [95% confidence interval]; 1.71 [1.30 – 2.26]), dyspnoea (1.36 [1.13 – 1.64]) and AFL with dyspnoea (2.10 [1.19–3.73]). Additionally, multivariate general linear models adjusted by age, sex, BMI, smoking status, and major comorbidities as well as subclinical respiratory dysfunction and spirometer at follow-up was used to calculate the adjusted risk ratios for the development of AFL, dyspnoea and AFL with dyspnoea. There were independent associations of high BNP with AFL. High BNP still had the positive risks for dyspnoea and AFL with dyspnoea although it did not reach statistical significance (Table S4).

**Supplementary Tables**

**Table S1 Characteristics of the subjects who were lost to follow-up.**

|  | Followed | Lost |
| --- | --- | --- |
| N | 5,086 | 779 |
| Age, y | 59 (± 9) | 60 (±11) ^*^ |
| Female, N (%) | 3,417 (67) | 469 (60) ^*^ |
| Height, cm | 159 (± 8) | 159 (± 9) |
| Weight, kg | 57 (± 10) | 58 (± 11) ^*^ |
| BMI, kg/m^2^ | 22.5 (± 3.0) | 22.8 (± 3.6) ^*^ |
| Smoking status, N (%)  Current  Former | 1,530 (30)  530 (10)  1000 (20) | 301 (39) ^*^  138 (18) ^*^  123 (21) |
| Pulmonary function test  FEV_1_, % pred  FVC, % pred  FEV_1_/FVC | 104 (± 14)  101 (± 14)  0. 82 (± 0.05) | 102 (± 15) ^*^  99 (± 15) ^*^  0.82 (± 0.05) |
| %FEV_1_ < 80%, N (%)  FEV_1_/FVC < LLN, N (%) | 200 (4)  45 (1) | 56 (7) ^*^  12 (2) |
| Comorbidities, N (%)  Hypertension  Diabetes  Cardiovascular disease  No. of comorbidities  1  2  3 | 1,193 (23)  326 (6)  222 (4)  1,207 (24)  229 (5)  24 (0.4) | 202 (26)  67 (9) ^*^  42 (5)  190 (25) ^*^  50 (6) ^*^  6 (1) ^*^ |
| All values are expressed as the mean (± SD), except categorical variables, which are expressed as N (%). AFL; airflow limitation, BMI; body mass index, FEV_1_; forced expiratory volume in 1 second, FVC; forced vital capacity.  ^*^; *P* < 0.05, comparing subjects who were lost to follow-up and those who underwent follow-up. | | |

**Table S2 Risk factors for the development of GOLD stage 2 or higher dyspnoea at 5 years.**

|  | RR  (95% CI) | Adjusted RR  (95% CI) |
| --- | --- | --- |
| Age ≥ 60 | 5.53 (1.93–15.8) | 2.27 (1.31–3.93) |
| Smoking status  Current vs. former | 1.41 (0.49–4.05) | 1.54 (0.53-4.53) |
| Former vs. never | 1.78 (0.76–4.15) | 1.34 (0.57–3.14) |
| Cardiovascular disease | 1.56 (0.38–6.53) | 1.04 (0.50–2.13) |
| %FEV_1_ < 80% | 18.7 (9.20–37.9) | 3.95 (2.73–5.71) |
| FEV_1_/FVC < LLN | 3.86 (0.54–27.7) | 3.61 (1.23–10.6) |

RR; risk ratio, CI; confidence interval, GOLD≥2; subjects who developed airflow limitation with %FEV1<80% at follow-up; FEV_1_; forced expiratory volume in 1 second, FVC; forced vital capacity, LLN; lower limit of normal.

**Table S3. Risk factors for the development of AFL (A), dyspnoea (B) and AFL with dyspnoea (C). FEV1/FVC was classified by** **the LLN defined by the GLI.**

A

|  | RR  (95% CI) | Adjusted RR  (95% CI) |  |
| --- | --- | --- | --- |
| Age ≥ 60 years | 2.24 (1.84–2.74) | 1.54 (1.36–1.73) |  |
| Female | 0.39 (0.33–0.47) | 0.67 (0.57–0.78) |  |
| Smoking status  Current vs. former | 1.54 (1.20–1.97) | 2.03 (1.45–2.84) |  |
| Former vs. never | 1.86 (1.51–2.29) | 1.07 (0.77–1.49) |  |
| BMI ≥ 25 kg/m^2^ | 0.77 (0.61–1.00) | 0.76 (0.66–0.88) |  |
| Hypertension | 1.31 (1.07–1.59) | 1.08 (0.95–1.22) |  |
| Diabetes | 1.08 (0.76–1.53) | 0.82 (0.66–1.02) |  |
| Cardiovascular disease | 1.19 (0.80–1.76) | 0.94 (0.74–1.20) |  |
| %FEV_1_ < 80% | 3.74 (2.96–4.73) | 2.18 (1.78–2.68) |  |
| FEV_1_/FVC < LLN (GLI) | 6.39 (4.26–9.59) | 6.10 (3.07–12.15) |  |
| SP-370 at follow-up ^a^ | 2.28 (1.61–3.22) | 1.59 (1.32–1.92) | |

B

|  | RR  (95% CI) | Adjusted RR  (95% CI) |
| --- | --- | --- |
| Age ≥ 60 | 1.41 (1.26–1.58) | 1.22 (1.13–1.31) |
| Female | 1.07 (0.95–1.21) | 1.19 (1.06–1.32) |
| Smoking status  Current vs. former | 1.41 (1.15–1.72) | 1.79 (1.37–2.34) |
| Former vs. never | 0.82 (0.75–1.01) | 0.94 (0.73–1.19) |
| Hypertension | 1.38 (1.23–1.55) | 1.14 (1.05–1.24) |
| Diabetes | 1.13 (0.92–1.39) | 0.99 (0.85–1.14) |
| Cardiovascular disease | 1.79 (1.48–2.16) | 1.43 (1.22–1.69) |
| %FEV_1_ < 80% | 1.27 (0.99–1.63) | 1.15 (0.96–1.37) |
| SP-370 at follow-up ^a^ | 1.08 (0.92–1.27) | 1.03 (0.94–1.14) |

C

|  | RR  (95% CI) | Adjusted RR  (95% CI) |
| --- | --- | --- |
| Age ≥ 60 | 2.98 (1.86–4.77)) | 1.72 (1.34–2.20) |
| Female | 0.48 (0.32–0.71) | 0.84 (0.63–1.11) |
| Smoking status  Current vs. former | 2.54 (1.44–4.49) | 3.32 (1.81–6.08) |
| Former vs. never | 1.37 (0.82–2.28) | 0.91 (0.47–1.75) |
| BMI ≥ 25 kg/m^2^ | 1.10 (0.68–1.79) | 0.97 (0.75–1.25) |
| Hypertension | 1.42 (0.93–2.17) | 1.07 (0.86–1.34) |
| Diabetes | 0.94 (0.42–2.14) | 0.75 (0.50–1.15) |
| Cardiovascular disease | 2.74 (1.48–5.05) | 1.53 (1.10–2.14) |
| %FEV_1_ < 80% | 3.73 (2.12–6.57) | 1.99 (1.48–2.66) |
| FEV_1_/FVC < LLN (GLI) | 5.22 (1.38–19.7) | 3.85 (1.72–8.64) |
| SP-370 at follow-up ^a^ | 2.38 (1.11–5.11) | 1.48 (1.02–2.16) |

**Table S3. Adjusted risk ratios for the development of AFL, dyspnoea and AFL with dyspnoea at 5 years.**

|  | AFL | Dyspnoea | AFL with dyspnoea |  |
| --- | --- | --- | --- | --- |
| Age ≥ 60 years | 1.51 (1.34–1.71) | 1.21 (1.12–1.30) | 1.69 (1.31–2.17) |  |
| Female | 0.67 (0.57–0.78) | 1.19 (1.07–1.32) | 0.84 (0.63–1.12) |  |
| Smoking status  Current vs. former | 2.05 (1.47–2.88) | 1.80 (1.38–2.36) | 3.36 (1.82–6.20) |  |
| Former vs. never | 1.09 (0.78–1.52) | 0.94 (0.74–1.19) | 0.91 (0.47–1.77) |  |
| BMI ≥ 25 kg/m^2^ | 0.76 (0.66–0.88) | 1.21 (1.11–1.33) | 0.97 (0.75–1.25) |  |
| Hypertension | 1.07 (0.95–1.22) | 1.13 (1.04–1.23) | 1.06 (0.85–1.33) |  |
| Diabetes | 0.82 (0.67–1.02) | 0.99 (0.85–1.14) | 0.76 (0.50–1.16) |  |
| Cardiovascular disease | 0.93 (0.73–1.19) | 1.42 (1.21–1.68) | 1.53 (1.09–2.14) |  |
| %FEV_1_ < 80% | 2.17 (1.77–2.67) | 1.14 (0.96–1.36) | 1.97 (1.47–2.65) |  |
| FEV_1_/FVC < LLN | 6.15 (3.09–12.22) | 0.96 (0.64–1.43) | 2.72 (1.44–5.12) |  |
| BNP > 40 pg/mL | 1.25 (1.04–1.50) | 1.14 (0.99–1.31) | 1.29 (0.95–1.76) |  |
| SP-370 at follow-up ^*^ | 1.58 (1.31–1.91) | 1.03 (0.93–1.14) | 1.46 (1.00–2.13) | |

Data are presented as adjusted risk ratios (95% confidence intervals).

AFL: airflow limitation; BMI: body mass index; FEV_1_: forced expiratory volume in 1 second; FVC: forced vital capacity; LLN: lower limit of normal.

*; spirometer used at follow-up.
